# Functional Neurological Disorder: A Case of Psychosomatic Paralysis Following Bereavement

**DOI:** 10.7759/cureus.78146

**Published:** 2025-01-28

**Authors:** Joseph T Luba, Benjamin KP Woo

**Affiliations:** 1 Psychiatry, Western University of Health Sciences, Pomona, USA; 2 Psychiatry, Olive View-University of California, Los Angeles (UCLA) Medical Center, Los Angeles, USA

**Keywords:** conversion disorder, functional neurological disorder, grief and neurological symptoms, la belle indifference, psychosomatic symptoms

## Abstract

Functional neurologic disorder (FND), alternatively known as conversion disorder, presents a unique diagnostic challenge due to its complex interplay of psychological, neurobiological, and social factors. This complexity is particularly evident in patients who experience significant psychological stressors, which may trigger or exacerbate symptoms. The following case report describes a 36-year-old woman who developed FND following the death of her father. This case highlights the diagnostic complexities of FND, particularly in patients with a prior psychiatric diagnosis. It underscores the importance of addressing the psychological factors underlying physical symptoms in such presentations.

## Introduction

Functional neurologic disorder (FND), previously referred to as conversion disorder, is a condition in which patients experience neurological symptoms such as paralysis, tremors, or non-epileptic seizures that cannot be explained by organic causes. Initially introduced in the Diagnostic and Statistical Manual of Mental Disorders (DSM) in its first edition, FND was historically categorized under psychoneurotic disorders and described as a manifestation of repressed psychological conflict [1]. The understanding of FND has since evolved, as reflected in subsequent editions of the DSM, including its current recognition in the Diagnostic and Statistical Manual of Mental Disorders, Fifth Edition (DSM-5) as a condition involving abnormal brain functioning without structural abnormalities [2,3].

Significant life events, whether positive or negative, can serve as critical triggers for the onset or exacerbation of symptoms in patients with FND. These "vital events" include negative experiences, such as losing a loved one, and positive yet overwhelming changes, such as a job promotion or marriage [2,3]. From a neurobiological perspective, FND is associated with functional abnormalities in brain regions involved in motor control, emotional processing, and the sense of agency, particularly the limbic system and motor cortex [2]. Clinically, FND often overlaps with other psychiatric or neurological conditions, necessitating careful assessment to differentiate it from disorders like schizophrenia or somatic symptom disorders [4]. A notable feature that can aid in the identification of FND is la belle indifference, a paradoxical lack of concern or emotional response to significant symptoms such as paralysis or seizures. While not universally present, this phenomenon reflects a disconnect between the patient’s symptoms and their emotional response, providing a helpful diagnostic clue in distinguishing FND from other conditions [5].

The Diagnostic and Statistical Manual of Mental Disorders, Fifth Edition, Text Revision (DSM-5-TR) outlines four key diagnostic criteria for FND. These include 1) the presence of one or more symptoms of altered motor or sensory function; 2) clinical findings that provide evidence of incompatibility between the symptoms and recognized medical conditions; 3) the symptoms are not better explained by another medical or psychiatric disorder; and 4) the symptoms cause significant distress or impairment in functioning [6]. Accurate diagnosis is crucial, as FND is more common than often recognized, with an estimated prevalence ranging from four to 12 per 100,000 individuals annually [7].

This case describes a 36-year-old female patient who lost the ability to walk following the death of her father, a significant negative life event. The patient exhibited marked physical, cognitive, and psychological decline, culminating in the onset of non-organic neurological symptoms. This presentation highlights the complex interplay of psychiatric illness, grief, and functional decline, ultimately resulting in her dependence on a wheelchair. The objective of this case presentation is to illustrate the diagnostic challenges of FND in the context of profound psychological trauma and a coexisting psychiatric history, emphasizing the importance of differentiating FND from other neurological and psychiatric conditions to inform effective treatment strategies.

## Case presentation

The patient was a 36-year-old unmarried woman with no children who was previously high-functioning socially, mentally, and physically. Mentally, her high functioning was evidenced by her academic achievements, graduating with a degree in English, and professionally, by her successful career as an accountant at an accounting firm. Socially, she engaged in meaningful relationships and community activities, playing varsity tennis in high school and club volleyball in college. Physically, she maintained an active lifestyle through competitive sports and recreational activities. She had been living independently but moved in with her father to care for him after he suffered a disabling stroke in 2018. The two were exceptionally close, and she became his primary caregiver as he experienced multiple strokes over the course of two years. 

Following her father’s death in 2020, the patient experienced profound grief, which quickly escalated to a series of psychiatric symptoms and physical decline. Over the next several months, she became increasingly isolated and withdrawn. She stopped attending work and refused to eat or leave her room. She began to believe that her father was visiting her in her room, and she removed the doorknob and barricaded the bedroom door with furniture. The patient's mother reported that she would lock herself in her room for weeks at a time, not eating or drinking, and screaming all day. She was even hospitalized with acute renal failure secondary to rhabdomyolysis due to her extreme dehydration and caloric restriction. These behaviors led to multiple psychiatric hospitalizations. Past discharge hospitalizations included anxiety disorder not otherwise specified (NOS) and schizophrenia with medication trials of Zoloft and Zyprexa. Notably, she had no family history of schizophrenia and had never used illicit drugs. 

Despite pharmacological therapy, the patient's functional status continued to decline. She became bedridden, non-ambulatory, and dependent on diapers and a wheelchair. Neurological workups, including EEG, brain MRI, and lumbar puncture, were all unremarkable. She was eventually transferred to a skilled nursing facility (SNF) in January 2021. At the SNF, her only medication was Zoloft. Notably, while only on Zoloft, she demonstrated significant improvement, including regaining some functional mobility. This suggests that her symptoms were less likely attributable to schizophrenia, which typically requires ongoing antipsychotic treatment for symptom stabilization. Her condition eventually improved, and she was able to get up from her wheelchair. Her family removed her from the SNF in August 2024, and she returned to live with her mother. However, after returning home, she stopped taking her medication, became resistant to care, increasingly isolated, refused to eat, and regressed to the point of urinating in trash bags.

In October 2024, the patient was admitted to our hospital on an involuntary hold for danger to self and inability to care for her basic needs after refusing food, water, and basic care for over 10 days. An altered mental status workup was performed, and she was medically cleared. Upon admission, she appeared withdrawn, unmotivated for self-care, wearing a diaper, and often disorganized in speech. She also refused to ambulate. According to the patient and her mother, she was not currently taking any medications. When asked about her mobility impairment, she consistently responded, "I use a wheelchair. My legs are tired."

A neurological exam revealed no focal deficits, with normal muscle strength of bilateral upper and lower extremities, intact sensation, and normal deep tendon reflexes throughout. Her lab results were mostly unremarkable, except for a prolactin level that was nearly five times the upper limit of normal, which in this case was likely related to kidney clearance. To assess her level of functioning in performing activities of daily living independently, we utilized the Katz Index [8]. The index assesses adequacy in performances of activities such as bathing, dressing, toileting, transferring, continence, and feeding. The patient scored 0 out of six, indicating severe dependence on basic activities. On the mental status exam, she demonstrated no delusions or hallucinations. She declined further diagnostic workups like a brain MRI and exhibited a lack of motivation to engage in her treatment plan. While not FDA-approved, to manage the patient's symptoms of depression and prolonged refusal to eat, she was started on a trial of Risperdal [9].

Physical therapy was consulted to evaluate her functional mobility. She was able to independently maneuver her wheelchair around the room, but she was unwilling to attempt walking in the hallway. When provided with a sturdy chair for support, she was able to stand but refused to walk, despite showing good strength in both hips and knees and having no significant muscular atrophy or contractures. When asked if she was interested in working with physical therapy, the patient repeatedly refused. Her inability to walk despite normal neurological findings, along with the inconsistency of her motor symptoms, aligns with the DSM-5-TR criteria for FND. The refusal to ambulate, despite good muscle strength and absence of muscular atrophy, points to a clear dissociation between physical ability and motor function, a hallmark of FND. Additionally, her apparent ambivalence toward her motor symptoms, illustrated by her lack of concern over her inability to ambulate and her repeated refusal to engage in physical therapy, points to the phenomenon of la belle indifference. This emotional detachment from significant symptoms is historically associated with FND and further supports the psychological rather than organic etiology of her condition [5]. In the absence of prominent psychotic symptoms, it became less clear that her condition was solely the result of a schizophrenia spectrum disorder.

The patient's treatment involved a multidisciplinary approach, addressing both her physical and psychological symptoms. Physical therapy was initially recommended to help her regain her mobility; however, her participation in physical therapy was minimal, as she frequently refused to engage in exercises aimed at restoring her walking ability. Pharmaceutical interventions were also implemented, including trialing Risperdal, as well as Cymbalta, which was introduced later to address her depressive symptoms [9]. Finally, recognizing the role of psychological trauma in the manifestation of her functional symptoms, cognitive-behavioral therapy (CBT) was recommended. This therapeutic approach aimed to address the underlying grief and psychological stressors that contributed to her functional decline, focusing on restructuring her thought processes and improving coping mechanisms. The combination of these treatments reflects the holistic approach necessary for managing FND, with an emphasis on addressing both the physical impairments and the psychological distress driving the disorder.

## Discussion

This case highlights the profound impact of psychological trauma, specifically grief, on physical and neurological functioning. The patient’s symptoms worsened after her father’s death, suggesting that unresolved grief and psychological distress may have played a pivotal role in triggering or exacerbating her functional decline. The description above exhibits that she fulfilled the diagnostic criteria for FND characterized by the DSM-5-TR. 

In the patient’s case, there was a notable presence of la belle indifference, a term used to describe a paradoxical lack of concern or emotional response to significant symptoms. While its diagnostic utility is controversial, in the context of FND, this phenomenon has historically been considered a characteristic feature [5]. Despite being non-ambulatory and entirely dependent on a wheelchair, she demonstrated minimal distress or urgency about her condition, as evidenced by her indifferent response to inquiries about her mobility. While la belle indifference is not universally present in FND cases, its occurrence in the patient, who exhibited minimal concern over her paralysis, further supports the diagnosis. The paradoxical emotional detachment from such severe symptoms often distinguishes FND from more distressing psychiatric conditions.

At the time of admission, schizophrenia was listed in the patient’s psychiatric history from a prior hospitalization at another facility; however, the presentation of non-organic motor symptoms, the temporal relationship to grief, and the absence of consistent and prominent psychotic features point to FND as the primary diagnosis. Differentiating negative symptoms of schizophrenia from FND involves careful clinical assessment, as both conditions can present with overlapping features such as reduced emotional expression and social withdrawal. Negative symptoms in schizophrenia are characterized by a reduction or absence of normal functions and behaviors. These include blunted affect, alogia, avolition, anhedonia, and asociality [4,10]. Functional neurologic disorder, on the other hand, involves neurological symptoms that are inconsistent with or cannot be fully explained by neurological or medical conditions [11]. Features of FND include motor symptoms such as tremors, dystonia, or gait abnormalities; sensory symptoms such as numbness or non-dermatomal sensory loss; and seizure-like episodes known as functional seizures, which are not associated with abnormal electrical activity in the brain [12]. 

To differentiate between negative symptoms of schizophrenia and FND, one must consider symptom onset and course, psychiatric history, neurological exam, and stress response [4,10,11]. The progression of her symptoms following her father’s death aligns more closely with the onset of FND rather than schizophrenia. Functional neurologic disorder symptoms, particularly functional impairments like non-ambulatory behavior, often have an acute onset linked to psychological stressors or traumatic life events [13-15]. In this patient’s case, her extreme grief response to her father’s death triggered her subsequent functional decline, including her isolation, refusal to eat, and non-ambulatory status. Negative symptoms of schizophrenia, such as lack of motivation and social withdrawal, tend to emerge more gradually and progressively over time, but her functional decline appears to have been more abrupt following her father’s death, suggesting a psychological stressor as the precipitating factor, which is more consistent with FND. 

Regarding psychiatric history, schizophrenia is typically characterized by positive symptoms such as hallucinations, delusions, and disorganized thinking, which were not predominant in her presentation. Throughout her hospital course, the patient exhibited no active delusions or hallucinations and demonstrated coherent, albeit unmotivated, behavior. Moreover, she did not exhibit these hallmark cognitive impairments commonly seen in schizophrenia, such as difficulty concentrating or deficits in abstract reasoning, which further weakens the argument for negative symptoms of schizophrenia. The absence of psychotic features, combined with her functional decline in the context of emotional trauma, strongly suggests FND over schizophrenia or agitated psychotic depression. Factitious disorders and malingering were considered in the differential diagnosis but were deemed unlikely based on the absence of external incentives, such as financial gain or avoidance of responsibilities, and the lack of evidence suggesting intentional deception. The patient's profound grief and functional decline, occurring in the context of a traumatic life event, strongly support a diagnosis of FND.

Furthermore, a hallmark of FND is the inconsistency and incongruence of symptoms, meaning that the symptoms vary over time or with different tasks and are not consistent with known neurological diseases [11]. This pattern was evident in her case. Despite her claims of leg weakness and non-ambulatory status, her physical examination demonstrated normal strength during specific tasks. During her neurological examination, she exhibited normal muscle strength of bilateral lower extremities, normal deep tendon reflexes, and intact sensation. However, she claimed that her legs were "tired" and refused to walk, relying on her wheelchair for mobility. When prompted to hold onto a sturdy chair, she was able to stand, showing that she had the physical capacity to engage in weight-bearing activities. Yet, when asked to walk, she refused, citing fatigue. This inconsistency between her ability to stand and her refusal to walk is typical of FND, where motor symptoms often fluctuate and do not align with organic neurological deficits. Additionally, the incongruence of her symptoms, where motor strength was preserved but the patient reported weakness, supports the diagnosis of FND. Such variability, seen with the ability to complete some tasks while refusing others, further distinguishes FND from neurological conditions where weakness is typically consistent across all tasks and settings.

Similar cases of FND triggered by significant life stressors, such as bereavement, have been reported, further emphasizing the role of psychological trauma in the development of psychosomatic symptoms [16]. This case contributes to the growing literature on the complexity of diagnosing FND in patients with coexisting psychiatric diagnoses. Understanding the intricate relationship between psychological trauma and physical symptoms is crucial for timely diagnosis and appropriate treatment. The suggestion of an underlying personality disorder, particularly a hysterical personality, is a possibility that could be explored further. However, during this episode of acute hospitalization, she declined personality testing, which limits our ability to assess her underlying personality traits in detail. In the future, psychological testing could provide valuable insights into her personality structure, coping mechanisms, and potential predispositions to FND. 

Successful management of FND requires a multidisciplinary approach involving rehabilitative and psychological therapies, with a multidisciplinary approach yielding the best outcomes [12]. The prognosis of FND varies. Early diagnosis and intervention are associated with better outcomes. However, the disorder can be chronic and disabling for some patients. Factors influencing prognosis include the duration of symptoms before diagnosis, the presence of comorbid psychiatric conditions, and the patient's engagement with treatment [12,17].

## Conclusions

This case underscores the intricate interplay between psychological trauma, psychiatric illness, and FND. The patient’s profound grief following the death of her father served as a pivotal trigger for the development of FND, manifesting as severe non-organic motor symptoms, including her inability to walk. Her symptoms, which persisted despite normal neurological findings and the absence of organic pathology, reflect the hallmark characteristics of FND. This highlights the critical role of psychological stressors in the pathogenesis of the disorder.

This case also emphasizes the diagnostic challenges posed by overlapping psychiatric conditions, such as schizophrenia, in patients presenting with unexplained neurological symptoms. The absence of consistent psychotic features, cognitive deficits, or gradual symptom progression commonly associated with schizophrenia supports FND as the primary diagnosis in this patient. Furthermore, her ambivalence toward her motor impairments aligns with the phenomenon of la belle indifference, reinforcing the psychological origins of her symptoms.

Importantly, this case illustrates the need for a comprehensive, multidisciplinary approach to FND, integrating physical rehabilitation, psychological therapies, and careful clinical assessment. Future evaluations, including personality testing, may provide further insight into underlying predispositions and coping mechanisms. This case serves as a reminder for clinicians to remain vigilant in recognizing FND, particularly in the context of significant psychological stress or trauma, to ensure timely and appropriate diagnosis and intervention. By addressing the complex interaction between mind and body, this approach offers the best potential for improved outcomes in patients with functional neurological symptoms.
